# Exploitation of Sugarcane Bagasse and Environmentally Sustainable Production, Purification, Characterization, and Application of Lovastatin by *Aspergillus terreus* AUMC 15760 under Solid-State Conditions

**DOI:** 10.3390/molecules28104048

**Published:** 2023-05-12

**Authors:** Ahmed M. A. A. Ramadan, Reda M. Shehata, Hussein H. EL-Sheikh, Fuad Ameen, Steven L. Stephenson, Sabry A. H. Zidan, Osama A. M. Al-Bedak

**Affiliations:** 1 Department of Botany & Microbiology, Faculty of Science, Al Azhar University, Cairo 11511, Egypt; micro_boy95@yahoo.com (A.M.A.A.R.); redamostafa.18@azhar.edu.eg (R.M.S.); husseinhosny.221@azhar.edu.eg (H.H.E.-S.); 2 The Regional Center for Mycology and Biotechnology (RCMB), Al Azhar University, Cairo 11511, Egypt; 3 Department of Botany & Microbiology, College of Science, King Saud University, Riyadh 11451, Saudi Arabia; 4 Department of Biological Sciences, University of Arkansas, Fayetteville, AR 72701, USA; slsteph@uark.edu; 5 Department of Pharmacognosy, Faculty of Pharmacy, Al-Azhar University, Assiut Branch, Assiut 71524, Egypt; 6 Assiut University Mycological Centre, Assiut 71511, Egypt

**Keywords:** *Aspergillus*, cholesterol, fungi, solid state, statins, anti-oxidant, antimicrobial

## Abstract

Using the internal transcribed spacer (ITS) region for identification, three strains of *Aspergillus terreus* were identified and designated AUMC 15760, AUMC 15762, and AUMC 15763 for the Assiut University Mycological Centre culture collection. The ability of the three strains to manufacture lovastatin in solid-state fermentation (SSF) using wheat bran was assessed using gas chromatography-mass spectroscopy (GC-MS). The most potent strain was strain AUMC 15760, which was chosen to ferment nine types of lignocellulosic waste (barley bran, bean hay, date palm leaves, flax seeds, orange peels, rice straw, soy bean, sugarcane bagasse, and wheat bran), with sugarcane bagasse turning out to be the best substrate. After 10 days at pH 6.0 at 25 °C using sodium nitrate as the nitrogen source and a moisture content of 70%, the lovastatin output reached its maximum quantity (18.2 mg/g substrate). The medication was produced in lactone form as a white powder in its purest form using column chromatography. In-depth spectroscopy examination, including ^1^H, ^13^C-NMR, HR-ESI-MS, optical density, and LC-MS/MS analysis, as well as a comparison of the physical and spectroscopic data with published data, were used to identify the medication. At an IC_50_ of 69.536 ± 5.73 µM, the purified lovastatin displayed DPPH activity. *Staphylococcus aureus* and *Staphylococcus epidermidis* had MICs of 1.25 mg/mL, whereas *Candida albicans* and *Candida glabrata* had MICs of 2.5 mg/mL and 5.0 mg/mL, respectively, against pure lovastatin. As a component of sustainable development, this study offers a green (environmentally friendly) method for using sugarcane bagasse waste to produce valuable chemicals and value-added commodities.

## 1. Introduction

Each year, agriculture generates five billion metric tonnes of lignocellulosic biomass, which includes barley bran, barley hay, bean straw, date palm leaves, rice bran, rice straw, sugarcane bagasse, wheat bran, wheat straw, fruits and vegetable wastes, wheat bran, and cotton leaf scraps. As a corollary, it is crucial to transform these wastes into meaningful industrial and commercial outputs while also limiting their negative environmental repercussions [1,2]. About half of the plant matter consists of lignocellulose, which is the most abundant green organic matter in the soil [3]. Lignocellulose consists of cellulose (35–50%), hemicellulose (20–35%), and lignin (15–25%), which are closely bound together by a combination of non-covalent and covalent bonds [4,5].

In adults, hypercholesterolemia is a metabolic disease that has been linked to cardiovascular morbidity and death in humans [6,7]. Lovastatin is an effective blood cholesterol-lowering medication for both humans and other animals. It is also a selective and robust competitive inhibitor of 3-hydroxy-3-methyl glutaryl coenzyme A (HMG-CoA). Lovastatin is a fungal secondary metabolite that was formerly known as mevinolin, monacolin K, and mevacor. It inhibits HMG-CoA reductase (E.C. 1.1.1.34), the first enzyme involved in cholesterol formation [7,8,9,10].

Lovastatin was the first statin to be authorized (in 1987) by the US Food and Drug Administration (FDA) to treat hypercholesterolemia [9,11]. According to reports, lovastatin possesses anticancer, immunomodulatory, and anti-inflammatory activities. Additionally, it is well documented to be effective in avoiding bone issues as well as undesirable neurological conditions [9,12,13,14]. More therapeutic uses of lovastatin have included the suppression of induced apoptosis, cellular proliferation, and necrosis in the context of blood malignancies, among other experimental scenarios. Additionally, it is utilized to treat conditions such as Alzheimer’s disease, coronary heart disease, certain bone problems, and low levels of tumor necrosis factors [9,15,16,17].

The polyketide pathway is used by fungi to manufacture the secondary metabolite lovastatin. Numerous species of fungi are recognized as lovastatin producers, including members of the genera *Aspergillus*, *Penicillium*, *Monascus*, *Paecilomyces*, *Trichoderma*, *Scopulariopsis*, *Doratomyces*, *Phoma*, *Pythium*, *Gymnoascus*, *Hypomyces*, and *Pleurotus* [9,18,19,20,21,22]. Of these, *Monascus ruber* and *Aspergillus terreus* are the leading and most targeted industrial manufacturers of lovastatin [11,23]. Surface fermentation, solid-state fermentation (SSF), and submerged fermentation (SmF) are some of the many fermentation techniques used to produce lovastatin. SmF is employed in batch and fed-batch modes for extensive commercial production [24,25]. In the SmF procedure, lovastatin might be produced using a nutrient-rich broth. Though several agro-wastes are utilized as substrates in the SSF process due to their affordability, eco-friendliness, long-term availability, and simplicity of downstream processing [16,17].

Sugarcane bagasse waste is generated throughout the globe at a rate of 54 million tonnes a year, with Egypt producing roughly 4.7 million tonnes of that total [1,26,27]. Due to this, finding a useful application for this waste is a study topic that promotes ecosystem conservation. Sugarcane bagasse, which is one of the lignocellulosic waste materials, has attracted a great deal of interest because of its potential as a bioadsorbent for the treatment of wastewater [28,29], a promising substrate for the production of ethanol [30,31,32,33] and a safe source for the production of enzymes using microorganisms. Therefore, the main goals of the present investigation were first to determine if three strains of *Aspergillus terreus* from Egyptian soil were capable of producing lovastatin from sugarcane bagasse waste using solid-state fermentation (SSF) and then to purify, characterize, and use the lovastatin that was generated from the most potent strain. 

## 2. **Results**

### 2.1. Morphological and Molecular Identification of the Strains of Aspergillus terreus

**Figure 1:**
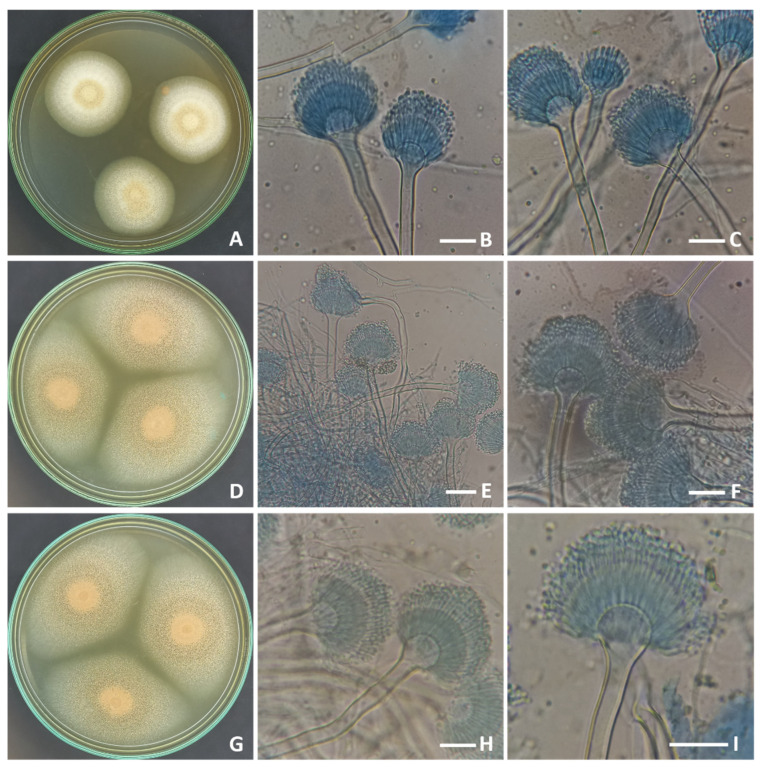
Seven-day-old colonies on MEA at 25 °C, conidiophores and conidial heads of (**A**–**C**), *A. terreus* AUMC 15760 (**D**–**F**), *A. terreus* AUMC 15762 (**G**–**I**), *A. terreus* AUMC 15763 (scale bars = 20 µm).

**Figure 2:**
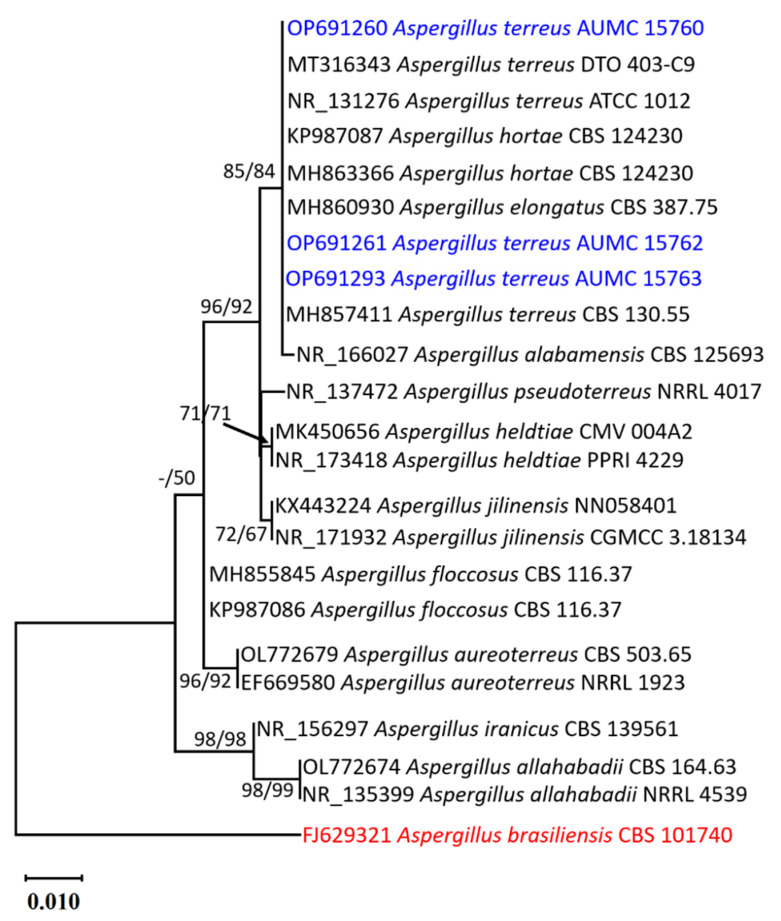
Maximum likelihood phylogenetic tree obtained from ML/MP analysis of ITS sequences of *A. terreus* AUMC 15760, AUMC 15762, and AUMC 15763 strains in this study (indicated in blue) compared to the most similar sequences of *Aspergillus*: section Terri in GenBank. Bootstraps (1000 replications) for ML/MP ≥ 50% are indicated above/below the respective nodes. *Aspergillus brasiliensis* CBS 101740 (indicated in red) was used as a tree-out group.

The morphological characteristics of the three strains of *Aspergillus* used in this study shared the identical features of *A. terreus* as producing cinnamon to orange-brown colonies with long and compactly columnar conidial heads borne on short and biseriate conidiophores (Figure 1). Phylogenetic analysis based on ITS sequencing was employed to confirm the identification of the strains. The final ITS data set contained 23 sequences, which produced 577 characters, of which 506 could be correctly aligned, 48 were counted as variable, and 22 were counted as informative. Tamura’s 3-parameter model using a discrete Gamma distribution (T92 + G) was the perfect model to represent the relationship between taxa. The Maximum Parsimony method yielded seven trees, the most parsimonious of which (Figure 2) had a tree length of 58, the highest log likelihood of −1013.83, a consistency index of 0.875000, a retention index of 0.945205, and a composite index of 0.827055, as shown.

### 2.2. Screening Activity of Lovastatin Production by Strains of A. terreus Using Wheat Bran in SSF

**Figure 3:**
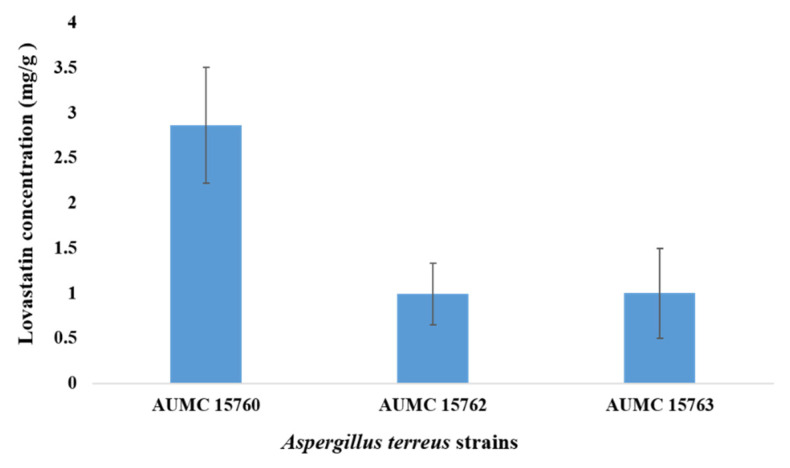
Lovastatin production by strains of *A. terreus* s on wheat bran under SSF at 30 °C.

Lovastatin production by strains of *A. terreus* utilizing wheat bran in SSF was tested. The three strains of *A. terreus* were evaluated quantitatively. The strain *A. terreus* AUMC 15760 could generate lovastatin at a significant high level of 2.86 ± 0.64 (*p* < 0.05) compared to the other two strains, AUMC 15762 and AUMC 15763, which produced 0.992 ± 0.34 and 0.998 ± 0.5 mg/g substrate, respectively (Figure 3).

### 2.3. GC-MS Detection of Lovastatin

The analysis was conducted for the qualitative detection of lovastatin in the methanolic extracts. The GC-MS chromatogram showed that the most abundant chemical compounds were represented by 10 peaks for the three strains (Figures S1–S3). These substances were recognized based on their molecular weight, retention time, and fragmentation pattern. At retention times of 21.672, 21.673, and 21.709 min for *A. terreus* AUMC 15760 (Table S1), AUMC 15762 (Table S2), and AUMC 15763 (Table S3), respectively, lovastatin was detected. In addition, a simvastatin compound was detected in the GC-MS chromatogram of *A. terreus* strains AUMC 15760 and AUMC 15763 at retention times of 28.669 and 28.687 min, respectively (Tables S2 and S3).

### 2.4. Lovastatin Production by A. terreus AUMC 15760 from Different Lignocellulosic Wastes

The current results showed that *A. terreus* AUMC 15760 could create lovastatin in varying amounts from the fermentation of the nine wastes utilized in SSF, with the exception of flax seeds. Sugarcane bagasse was the most profitable substrate when compared to the other wastes that were evaluated. According to spectrophotometric measurements, this type of waste produced the most lovastatin (3.9 ± 0.24 mg/g), while the remaining wastes showed modest levels of lovastatin production, ranging from 2.86 ± 0.6 mg/g in wheat bran to 0.2 ± 0.07 mg/g in barley bran. Furthermore, flax seed did not yield lovastatin.

### 2.5. Optimization of Lovastatin Production by A. terreus AUMC 15760

**Figure 4:**
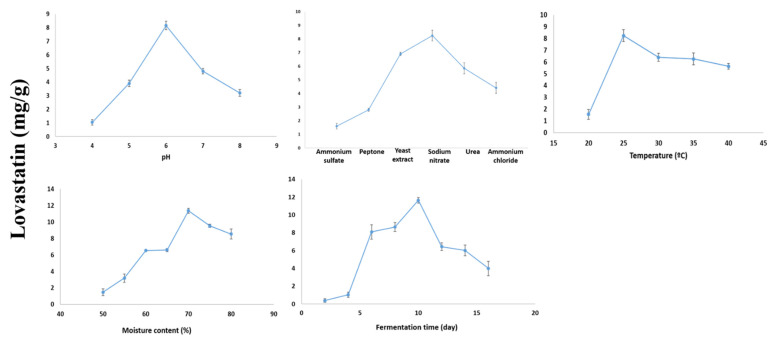
Optimization of fermentation conditions for lovastatin production by *A. terreus* AUMC 15760 from sugarcane bagasse under SSF.

*Aspergillus terreus* AUMC 15760 was used in SSF to produce lovastatin from sugarcane bagasse as a substrate. It was able to ferment sugarcane bagasse at a range of pH values (4–8) and create lovastatin at varying concentrations. The fungus could generate the greatest amount of lovastatin (8.16 ± 0.32) mg/g substrate at pH 6.0 with no significant difference (*p* > 0.05). While ammonium sulfate recorded the lowest level of lovastatin (1.6 ± 0.2 mg/g), sodium nitrate was able to raise the production of lovastatin to 8.25 ± 0.4 mg/g (*p* > 0.05). The fungus generated a significant amount of lovastatin (8.24 ± 0.5 mg/g) at 25 °C (*p* < 0.05), and when the moisture content was set at 70%, the highest level of lovastatin production (11.35 ± 0.31 mg/g) was significantly greater (*p* < 0.05). After 10 days of incubation, the yield increased to 11.64 ± 0.3 mg/g. The last incubation period was accompanied by a decrease in lovastatin production to 6.44 ± 0.43, 6.02 ± 0.6, and 4.0 ± 0.8 mg/g. Lovastatin production also occurs on the 12th, 14th, and 16th days of incubation (Figure 4). In general, it can be said that temperature and moisture content are the only two factors that significantly affect the results.

### 2.6. HPLC Analysis of Methanolic Extracts

**Figure 5:**
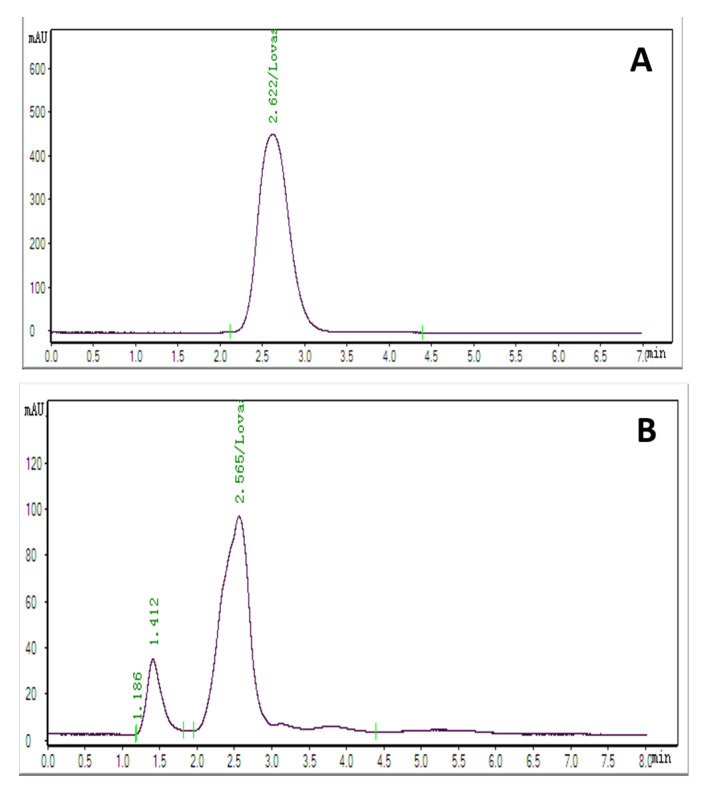
HPLC chromatogram of lovastatin production by *Aspergillus terreus* AUMC 15760 in SSF from sugarcane bagasse: (**A**) standard lovastatin and (**B**) lovastatin produced from sugarcane bagasse.

Lovastatin was synthesized and HPLC-analyzed under the optimum solid-state conditions following eight days of fermentation at pH 6.0 and 25 °C, utilizing sodium nitrate as a nitrogen source at 70% moisture content. It was noted for the sugarcane bagasse methanolic extract that *A. terreus* AUMC 15760 was used as its carbon source. When compared to the standard lovastatin (2.622 min) (Figure 5A), the fungus was able to produce lovastatin in large amounts (18.2 mg/g), with an average retention time of 2.562 min (Figure 5B). According to current data, the HPLC-accurate estimation of lovastatin concentration was found to be 18.2 mg/g.

### 2.7. Production and Purification of Lovastatin by Column Chromatography

*A. terreus* AUMC 15760 was capable of fermenting the sugarcane bagasse used in this investigation in SSF under ideal circumstances, yielding 31.0 g of the crude residue after drying the methanol extract under reduced pressure. Twelve fractions (Fr.1–Fr.12) were obtained using the solvent system of 0–100% gradients of EtOAc in n-hexane from the VLC column. The presence of lovastatin was verified by TLC on fraction No. 8 (Fr. 8), which was eluted with EtOAc:n-hexane (80:20) and produced 460 mg of crude drug after the evaporation of the solvent. The n-hexane:EtOAc (20:80) solvent system was used for open column chromatography (1.0 cm × 100 cm) to further purify the resulting fraction (460 mg), yielding ten sub-fractions (Fr. 8-S1–Fr. 8-S10). Of these, Fr. 8-S5 had the greatest lovastatin content (45 mg). Fr. 8-S5 was extra-purified using a 0.5 cm × 25 cm column and eluted with n-hexane:acetone (50:50), yielding 6.2 mg of pure lovastatin.

### 2.8. LC-MS/MS and HR-ESI-MS Analysis

A portion of the lovastatin-containing fraction (Fr.8-S5) was subjected to LC-MS/MS analysis. A significant peak was observed on the LC chromatogram at a retention time of 15.95 min (Figure S4). Two significant mass ion peaks at *m*/*z* 405.29 and 427.26 were observed in its mass spectrum. By fragmenting this peak using LC-MS/MS, the fragments 285.21, 303.21, 225.21, and 199.17 were distinctively recorded. The HR-ESI-MS of the purified lovastatin showed a molecular ion peak [M + Na]^+^ at *m*/*z* 427.2455 calculated for C_24_H_36_O_5_Na (427.2460), indicating seven degrees of unsaturation (Figure S5). 

### 2.9. NMR

**Figure 6:**
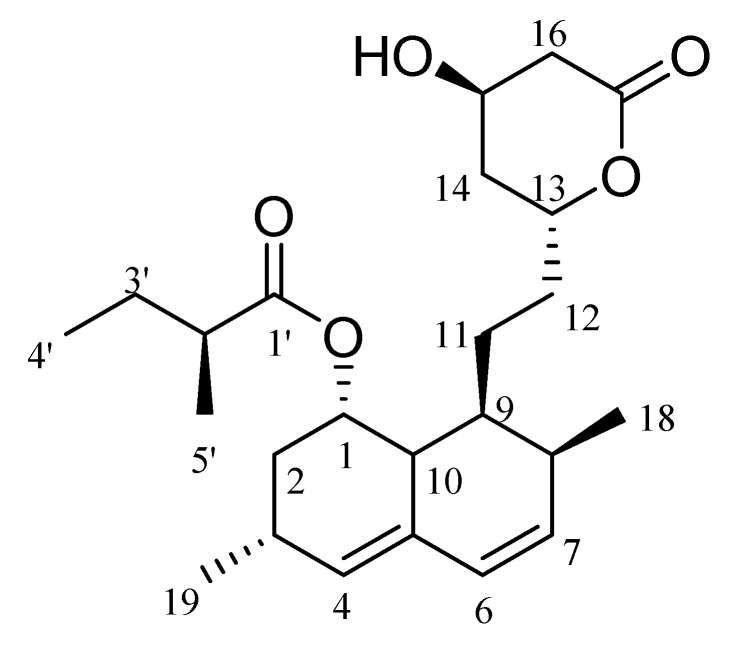
Chemical structure of lovastatin produced by *A. terreus* AUMC 15760 from sugarcane bagasse in SSF.

For final confirmation of the lovastatin produced in this project, the sub-fraction Fr.8-S5 (45 mg) was subjected to different chromatographic steps to isolate its major compound, lovastatin (Figure 6). It was isolated as a white powder with an optical activity of +315 (*c* = 4 mg/mL, acetonitrile). The ^1^H and ^13^C-NMR spectra revealed the presence of 24 carbon functionalities categorized as four methyls, six methylenes, eleven methines, and three quaternary carbons. Detailed ^1^H and ^13^C-NMR data are listed in Table 1 and Figures S6 and S7.

### 2.10. Antioxidant Activity of the Pure Lovastatin Produced by A. terreus AUMC 15760 

**Figure 7:**
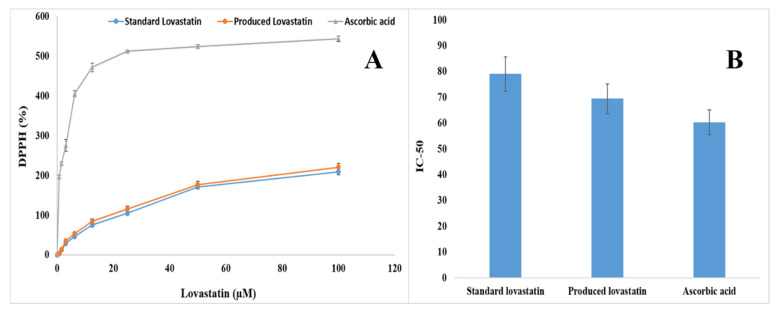
(**A**) Antioxidant activity (DPPH %) and (**B**) IC_50_ of the purified lovastatin produced by *A. terreus* AUMC 15760 (this study) compared to the standard lovastatin drug.

The hydrogen-scavenging capabilities of lovastatin synthesized by *A. terreus* AUMC 15760 were further examined using a DPPH test, and the free radical scavenging activity was reported as an IC_50_ value. According to current data, increasing the sample concentration led to an increase in DPPH activity (Figure 7A), and the IC_50_ value (69.536 ± 5.73 µM) of the produced lovastatin and the standard value (79.1 ± 6.66 µM) were almost equal. However, both IC_50_ values were slightly less than the value for ascorbic acid activity (60.3 ± 4.77 µM), indicating no significant difference (*p* > 0.05) (Figure 7B).

### 2.11. Antimicrobial Ability of the Purified Lovastatin Produced by A. terreus AUMC 15760

In this project, the antimicrobial activity of purified lovastatin was assessed against three strains of *Candida* and three strains of bacteria (one Gram-negative and two Gram-positive). The current results showed that the purified lovastatin had a MIC of 1.25 mg/mL and resulted in a significant inhibition of 11.5 ± 0.7 and 11.4 ± 0.4 mm for *S. aureus* and *S*. *epidermidis*, respectively (Table 2; Figure S8). The impact of the purified lovastatin was quite comparable to the antibiotic standard (P/T), particularly for *S. epidermidis*, and it was only about two-thirds as effective against *S. aureus* as the standard. On the other hand, the MIC for lovastatin in its purified form was 2.50 mg/mL, with an inhibition of 9.2 ± 0.2 mm for *C. albicans* that roughly equated to 40% of the Nystatin action. The drug had a MIC of 5.0 mg/mL when employed against *C. glabrata*, which resulted in inhibitions of 8.7 ± 0.4 mm (Table 2; Figure S8), denoting around 40% as the Nystatin effect. However, *C. krusei* and *E. coli* were unaffected by the medication. 

## 3. Discussion

Numerous fungi can generate various types of secondary metabolites, including enzymes, lipids, fats, and substances of great medical and industrial potential, such as lovastatin, a superior substrate for the hyper-production of the cholesterol lowering pharmaceutical drug [34,35]. One of these fungi is *A. terreus,* which is widely recognized for producing the compound lovastatin by submerged fermentation (SmF) or solid-state fermentation (SSF), both of which make use of lignocellulosic biomass and different synthetic media [9,17,36,37,38,39].

The ingredients in food or animal feed compete with these substrate resources, which might be costly. However, significant quantities of agro-industrial waste are generated globally, particularly in tropical nations. The majority of these agricultural wastes are burned for disposal, which has serious negative effects on the environment. It is just a small portion of it that is fed to ruminant cattle as roughage. However, these biomasses might promote the growth of microorganisms and the creation of biomaterials [1,40]. Additionally, the use of such materials in SSF by fungi in the pharmaceutical production of some key metabolites can reduce the use of dangerous chemicals that have a detrimental effect on the environment.

Consequently, the present investigation was carried out to determine the ability of strains of *A. terreus* isolated in this project to utilize some lignocellulosic residues as carbon sources in SSF and produce lovastatin. Three strains of *A. terreus* were selected, and these could ferment nine lignocellulosic residues with varied abilities and produce lovastatin. In this regard, different efforts were made to substantially boost lovastatin production. The selection of fermentation modes and the optimization of the fermentation medium have been the main areas of focus for a number of previous studies [41,42,43,44,45,46,47,48,49,50,51,52]. The majority of investigations have focused on *A. terreus* ATCC 20541 [53] and *A. terreus* ATCC 74135 [54], while others explored wild strains of *A. terreus* [24,55], *A. terreus* ATCC 20542 [39], and mutant strains of *A. terreus* [56,57]. 

The most potent strain, *A. terreus* AUMC 15760, produced the most lovastatin. Similarly, lovastatin has been produced from *Aspergillus terreus* ATE-120 [17], *Aspergillus terreus* ATCC 20542 [39,58], *Aspergillus niger* NRRL 595, *Rhizopus oligosporus* NRRL 2710, *Aspergillus fumigatus*, *Penicillium citrinum* [22], and *Penicillium funiculosum* NCIM 1174 [59].

In the present study, sugarcane bagasse showed better results in the screening experiment than the remaining residues used (barley bran, bean hay, date palm leaves, flax seed, orange peels, rice straw, soybean, and wheat bran). Sugarcane bagasse is a complex byproduct of the sugarcane industry. The chemical composition of sugarcane bagasse is cellulose (50%), hemicellulose (25%), and lignin (25%) [17,34], making it a promising substrate to be economically utilized in biotechnological processes.

In this study, *A. terreus* AUMC 15760 exploited sugarcane bagasse as a substrate, and after adjusting fermentation parameters such as pH, nitrogen supply, temperature, fermentation length, and moisture content, a significant concentration of lovastatin (18.2 mg/g) was produced. In the HPLC analysis, the ideal conditions of pH 6.0, 25 °C, 10 days, sodium nitrate, and 70% moisture content led to the greatest lovastatin production (18.2 mg/g). In this regard, *A. flavipes* BICC 5174 employed wheat bran as a substrate at pH 5.0 and produced lovastatin levels of 13.49 and 16.65 mg/g, respectively, in stagnant beds and aerated agitated beds [60], which were lower than those in those observed in this research. It has been stated that sodium nitrate is required to enhance the manufacturing of statins and that supplies of carbon and nitrogen are essential for the production of lovastatin [22,54,61]. Lovastatin production has already been shown to be significantly controlled by pH [62], due to the fact that the number of chemicals that are carried across the physiological membrane is significantly influenced by pH [63].

At day 10 of the fermentation process, the *Aspergillus terreus* strain in this study was in its log phase and began the fermentation process by creating secondary metabolites in an escalating pattern, with the largest output of lovastatin occurring. The production of different metabolites starts to stabilize at this point and continues through the stationary growth phase. The output of metabolites begins to decrease when the organism enters its lag phase and the cells begin to age. Samiee et al. [64] and Dewi et al. [65] reported similar findings on the lovastatin production by *A. terreus*, which peaked after 7 days of fermentation under SSF. Other researchers discovered that lovastatin production by different fungi was best achieved during incubation durations of 6–10 days [66]. While SSF using oat, wheat, and wheat bran produced the most lovastatin on the eleventh day of growth in the *A. terreus* 4, *A. terreus* 20, and *Aspergillus* sp. no. 76 strains, respectively [67,68]. According to Pansuriya and Singhal [69], *A. terreus’s* lovastatin output in SSF increased up to day 3 of fermentation before declining further [70].

*Aspergillus terreus* AUMC 15760 in this study yielded 18.2 mg/g, which was significantly higher than that produced by *A. flavus* A13 (0.585 mg/g), *A. fumigatus* A61 (0.658 mg/g), and *A. terreus* A50 (10.23 mg/g), respectively, when used with 1:1 wheat bran and oat bran as a substrate in SSF [36], and higher than that produced by *A. terreus* UV 1718 (2.914 ± 0.03 mg/g) using wheat bran in SSF [69] or that produced by *Penicillium funiculosum* NCIM 1174 (5.3 mg/g) under SSF when using wheat bran as a substrate [59].

Column chromatography was used in the current to purify the lovastatin that was produced, and LC-MS/MS, NMR, and HR-ESI-MS were applied to further confirm the purity of that compound. The information proved it to be lovastatin in its lactone form. Our conclusions are further supported by its optical rotation values. These outcomes were supported by previous findings [71,72].

The fraction that contained the most lovastatin showed two molecular ion peaks at *m*/*z* 405.29 [M + H]^+^ and 427.26 [M + Na]^+^ in the mass spectrum, which are consistent with [M + H]^+^ and [M + Na]^+^, respectively. They were compatible with the molecular formula of lovastatin in its lactone form (C_24_H_36_O_5_). For more confirmation of this placement, this peak was subjected to fragmentation using LC-MS-MS. Interestingly, a distinctive lovastatin peak was found. The values 285.21, 303.21, 225.21, and lastly 199.17 are all consistent with the fragments [M + H-C_5_H_10_O-H_2_O]^+^, [M + H-C_5_H_10_O]^+^, [C_17_H_20_ + H]^+^, and [C_15_H_18_ + H]^+^, respectively. These outcomes were supported by the findings reported by [73,74].

NMR and HR-ESI MS were used as the final validation of the lovastatin generated in the present study. The ^1^H NMR spectrum revealed the presence of four methyl groups, three of which were secondary methyls that resonate at ppm values of 0.94 (CH_3_-18), 1.10 (CH_3_-19), and 1.12 (CH_3_-5′), in addition to a primary methyl at ppm 0.93 (CH_3_-4′), which was confirmed by ^13^C-NMR signals [14.1, 23.3, 16.6, and 12.1 ppm], respectively. The olefinic protons resonating at 5.52 and 6.00 ppm and the four carbon signals resonating at 130.3, 129.5, 129.8, and 133.9 ppm were used to compute two double bonds. These findings are consistent with those of [75], who employed high-speed countercurrent chromatography (HSCCC) to characterize mevinolinic acid from *Monascus purpureus*. The ^13^C-NMR also revealed two downfield carbonyl carbons at 173.4 and 178.2 ppm and three distinctive signals at 69.5, 78.0, and 63.3 ppm assigned to three oxygenated carbons. Five methines and six methylenes were also recorded [71]. Comparing these data (HR-ESI-MS, ^1^H and ^13^C-NMR, optical density) with literature confirmed the structure of the pure isolate as lovastatin. 

The lovastatin obtained in this study was shown to exhibit DPPH activity based on the available data, and the IC_50_ value was essentially identical to the standard lovastatin. However, neither of the IC_50_ values was considerably lower than that of ascorbic acid activity. In this context, when lovastatin from *Pleurotus ostreatus* was investigated, it was demonstrated to have an antioxidant effect that prevents the synthesis and generation of free radicals as well as actively disrupts them, lowering the risk of cancer and cardiovascular disease [76]. 

Reactive oxygen species (ROS), which arise as a result of hypercholesterolemia, contribute to oxidative stress by changing other biological macromolecules [77,78]. Reduced glutathione (GSH), catalase, and superoxide dismutase (SOD) are significant defensive antioxidants that scavenge oxygen free radicals and thus defend against atherosclerosis [79]. An essential role in the body’s defense against free radicals, peroxides, and a wide range of xenobiotics and carcinogens is played by the endogenous antioxidant glutathione [80]. Lovastatin prevents ROS from entering the cell membrane and even from being scavenged [81,82]. The prevention of superoxide anion production is another mechanism behind the antioxidant action of lovastatin. Additionally, it keeps intracellular SOD functioning and eventually prevents inflammation [78,83]. 

In comparison with Nystatin and Piperacillin/Tazobactam, pure lovastatin significantly inhibited the growth of *C. albicans*, *C. glabrata*, *S. aureus*, and *S. epidermidis* in the current project. According to previous research, species of *Aspergillus* are a rich source of secondary metabolites with intriguing biological properties, such as antibacterial activity [84,85]. The antifungal property of lovastatin is well known; it prevents the development of representatives of several fungal genera, such as *Saccharomyces cerevisiae*, *Candida* spp., *Aspergillus* spp., and *Cryptococcus* spp., by inhibiting the enzyme HMG-CoA reductase, which depletes ergosterol, the fungal version of cholesterol [9,86,87]. HMG-CoA reductase is used by Gram-positive bacteria like *S. aureus* and *Enterococcus faecalis* for their mevalonate-dependent isoprenoid production [82,88]. Therefore, bacterial HMG-CoA reductase appears to be a promising target for statins. Similar to atorvastatin, simvastatin dramatically reduced *S. aureus*, *E. coli*, and *Enterobacter faecalis* growth [89]. It is consistent with prior research that found atorvastatin and simvastatin suppressed the development of an isolate of *Aspergillus fumigatus* and that lovastatin and simvastatin can inhibit the growth of *Aspergillus* spp. [90]. Simvastatin’s capacity to induce a post-antibiotic effect (PAE) is an intriguing discovery since it suggests that the drug may be able to inhibit bacterial growth for a longer length of time even at doses below the MIC. Since drugs that do not exhibit this post-effect require less frequent treatment than antimicrobial treatment does, PAE is a crucial characteristic that influences antibiotic regimens [91]. Simvastatin’s antibacterial activity may vary depending on whether the bacteria are Gram-positive or Gram-negative due to structural variations between the two types of bacteria. Gram-positive and Gram-negative bacteria vary in their peptidoglycan (cell wall) structures, and this difference may prevent Gram-positive bacteria from acting as an antibiotic agent (peptidoglycan is thicker) [92,93].

To withstand stressful situations, certain bacteria require a molecule such as a sterol. Statins may deprive bacteria of a metabolite essential for the integrity of their membranes, which cholesterol efficiently replaces. Bacteria can withstand the damaging effects of statins in this way [94]. It has been convincingly demonstrated that cholesterol can take the place of various membrane stabilizing chemicals, and there is a connection between a sterol and the antibacterial activity of statins in light of this evidence. Statins may block a step in the isoprenoid biosynthesis pathway that is essential for maintaining membrane integrity; this step is replaced by cholesterol, which shields bacteria from statin toxicity. Statins may have dual therapeutic advantages by directly eliminating bacteria and reducing the amount of host cholesterol that is available for bacterial development [89].

## 4. Materials and Methods

### 4.1. Chemicals and Materials

Organic solvents were acquired from El-Nasr Pharmaceutical and Chemical Co. (ADWIC), Cairo, Egypt, and utilized for extraction, fractionation, and column chromatography. TLC-precoated plates (F_254_) and silica gel 60 for column chromatography (70–230 and 230–400 mesh) were acquired from Merck (Darmstadt, Germany). Standard lovastatin was purchased from Sigma-Aldrich Chemical Co. (St. Louis, MO, USA).

### 4.2. Strain Isolation and Preservation

The strains involved in this work were isolated from desert soil collected from Sohag and Aswan governorates, Egypt. The acquired samples were kept in sterile plastic bags and quickly transferred to the laboratory of the Assiut University Mycological Centre (AUMC) for isolation of fungi using the dilution plate method [95]. Before filling Petri plates with Czapek’s Dox agar (CzA), suitable dilutions of the soil solution were added. The cultures were then incubated for two weeks at 25 °C. To obtain pure cultures of the fungus, the developed colonies were purified on CzA using the single spore isolation technique [96].

### 4.3. Morphological and Molecular Identification of the Aspergillus terreus Strains

Fungal strains were inoculated on CzA, malt extract agar (MEA), and Czapek yeast autolysate agar (CYA) [97]. Microscopic features on MEA were examined in lactophenol cotton blue after seven days of incubation at 25 °C, and the strains used in this study were identified according to their macroscopic and microscopic characteristics [98]. The three *Aspergillus* strains obtained were maintained in the culture collection of the Assiut University Mycological Centre as AUMC 15760, AUMC 15762, and AUMC 15763. Fungal DNA was isolated [99], and the PCR reaction was performed using SolGent EF-Taq [100]. ITS1 and ITS4 universal primers were used for amplification of the ITS region [101]. DNASTAR (version 5.05) was used to produce contiguous sequences of the species of *Aspergillus* included in this study. An outgroup sequence for *Aspergillus brasiliensis* CBS 101740 (FJ629321), three sequences for *Aspergillus* spp. (AUMC 15760, AUMC 15762, and AUMC 15763 in this work), and 19 sequences from the genus *Aspergillus* retrieved from GenBank made up the 23 sequences in the total ITS dataset included for phylogenetic analysis. All sequences were aligned using MUSCLE [102], and the alignment gaps and weak uninformative characters were manually optimized. MEGA X (version 10.2.6) was used to conduct maximum-likelihood (ML) and maximum-parsimony (MP) phylogenetic analyses [103], and the robustness of the most parsimonious trees was evaluated by 1000 replications [104]. Utilizing Modeltest 3.7’s Akaike Information Criterion (AIC), the optimum nucleotide substitution model for ML analysis was identified [105]. 

### 4.4. Screening of Lovastatin Production by Strains of A. terreus under SSF 

Ten grams of wheat bran were separated into separate Erlenmeyer flasks (500 mL). The residue was then moistened with citrate buffer (pH 5.0) until it was 80% wet. *Aspergillus terreus* strains AUMC 15760, AUMC 15762, and AUMC 16763 in cultures that were each seven days old and contained 1.5 × 10^8^ spore/mL were used to generate a spore suspension (5 mL) for each flask. The flasks were next incubated for seven days at 30 °C.

#### Extraction of Lovastatin

After the fermentation process was completed, the solid substrate and the mycelial mat were then dried at 60 °C for 24 h, crushed, and separately extracted by shaking in 50 mL of methanol at 180 rpm for 2 h. The cell-free supernatant was eventually collected by centrifugation at 10,000 rpm at 4 °C and employed as a source of lovastatin. 

### 4.5. GC-MS Analysis

This analysis was carried out at the Analytical Chemistry Unit (ACAL), Faculty of Science, Assiut University, Egypt, for the determination of lovastatin produced. Prior to the GC/MS analysis, 0.5 g of the sample was dissolved in 5 mL of methanol and then centrifuged at 10,000 rpm for 15 min at 5 °C. The analyzed fraction was subjected to a GC-MS apparatus (7890A-5975B; Thermo Scientific GC/MS; model ISQ; Waltham, MA, USA). The equipment has a HP-5MS Capillary Standard nonpolar column with dimensions of 30 mm × 0.25 mm × 0.25 μm. The cycle was as follows: equilibration time 0.5; max temperature 280 °C; oven program on at 120 °C for 5 min; 30 °C/min rose to 265 °C for 25 min; then 50 °C/min rose to 280 °C for 5 min; run time 48 min; post run 260 °C for 2 min; flow program 0.5 mL min for 10.9 min and then 1 mL min for 30 min; MS source 230–250 °C; and MS Quad 150–200 °C. The most effective strain of *A. terreus* was chosen to produce lovastatin under SSF, utilizing a variety of lignocellulosic wastes as a carbon source.

### 4.6. Lovastatin Production by A. terreus AUMC 15760 from Different Lignocellulosic Wastes

The substrates for fermentation were barley bran (BB), bean hay (BH), date palm leaves (DPL), flax seed (FS), orange peels (OP), rice straw (RS), soybean (SB), sugarcane bagasse (SCB), and wheat bran (WB). They were acquired at local markets in Egypt’s Assiut Governorate. They were washed in tap water to get rid of dust and other impurities before being ground down to a size of 500 µm. The samples were thoroughly filtered after being treated with 1% NaOH, rinsed with tap water to finish the pretreatment procedure, and then dried at 65 °C [106]. The analyzed agro-industrial residues were divided into 10 g portions and placed in separate Erlenmeyer flasks (500 mL). The residues were then further soaked with citrate buffer (pH 5.0) until 80% moist. Each flask received 5.0 mL of a spore suspension that was prepared from an *A. terreus* AUMC 15760 culture that was seven days old and contained 1 × 10^8^ spore/mL. The flasks were then incubated at 30 °C for seven days. Lovastatin was extracted as described above.

### 4.7. Optimization of Lovastatin Production by A. terreus AUMC 15760 from Sugarcane Bagasse 

Under a single factor at a time (OFAT) condition, pH (4.0, 5.0, 6.0, 7.0, and 8.0) at 30 °C, temperature (20, 25, 30, 35, and 40 °C) at the optimum pH, nitrogen source (peptone, yeast extract, sodium nitrate, ammonium chloride, ammonium sulfate, and urea; each at 0.2%) at the optimal pH and temperature, moisture content (50, 60, 70, and 80%) at the optimal pH, temperature, and nitrogen supply, and incubation time (2, 4, 6, and 16 days) using the optimum conditions, were adjusted to optimize the yield of the *A. terreus* AUMC 15760. The experiments were carried out using portions of 10 g of sugarcane bagasse in 500 mL Erlenmeyer flasks. Each flask was individually spore-inoculated with a 2.0% (*v*/*v*) spore suspension produced from 7-day-old *A. terreus* AUMC 15760. Finally, lovastatin was extracted, as already mentioned. According to the Markopoulou and Koundourelllis [107] method, the content of lovastatin in the culture supernatants of the *A. terreus* strains was measured using a UV-visible spectrophotometer (T80+; Leicester; UK). The reaction was conducted in a separate mixture of 1.0 mL of the supernatants and 1.0 mL of 1.0% trifluoracetic acid. The mixture was then incubated at 30 °C for 10 min. After being diluted ten times with methanol, the absorbance of the latter was quantitatively determined at 238 nm. Using the lovastatin standard curve, the lovastatin concentration was calculated per gram of dry substrate according to Equation (1):

(1) $$\mathrm{Lovastatin}\;\mathrm{concentration}\;=\;\frac{Lovastatin\;concentration\;(mg/mL)}{Substrate\;weight\;\left(g\right)\;\times \;Dilution\;Factor}\;\cdot \;mg/g$$

### 4.8. HPLC Assay of the Produced Lovastatin by A. terreus AUMC 15760

Lovastatin concentration was assayed by the HPLC method at the HPLC unit, the Regional Center for Mycology and Biotechnology, Al-Azhar University, Cairo, Egypt. Extracts were dried and concentrated under reduced pressure. A HPLC system (E-Chrom Tech; Model LC 1620 Aliquid chromatograph) with UV detector was used for this assay. Ten µL of the sample was injected into the C-18: Shodex C-18-120-5 4E (250 × 4.6 mm) column using the mobile phase of an acetonitrile: 1.1% orthophosphoric acid (95:5; *v*/*v*) at a flow rate of 1.3 mL/min. With the use of PA Station 2015 ChemStation (version 2.0) software, lovastatin peaks at 238 nm were monitored in comparison to the standard lovastatin (Sigma-Aldrich), and the lovastatin concentration was calculated per gram of dry substrate (gds).

### 4.9. Production of Lovastatin from Sugarcane Bagasse by A. terreus AUMC 15760 in SSF

The sugarcane bagasse residue was utilized in SSF to prepare lovastatin using *A. terreus* AUMC 15760. To do this, 150 g were split into 10 g parts, and each portion was placed individually into a 500 mL Erlenmeyer conical flask. *Aspergillus terreus* AUMC 15760 was used as the source of the 1.5 × 10^8^ spore/mL spore suspension to inoculate the flasks. The flasks were maintained under optimized production conditions. After the incubation period, the contents of the flasks were 60 °C oven-dried, gently crushed, and collected in 750 mL of methanol by shaking at 180 rpm for two hours. By centrifuging at 10,000 rpm and 4 °C, the cell-free supernatant was eventually recovered. Using a rotary evaporator (Heidolph: Model reddot winner 2020; Germany), the supernatant was then concentrated at 40 °C under reduced pressure. Following that, a freeze dryer (VirTis: Model 6 KBTES-55, NY, USA) was used to lyophilize the resulting residue to be used in the purification procedure.

### 4.10. Purification of Lovastatin

Prior to the purification process, the residue was combined with the same weight of silica gel 60 powder and a small amount of methanol to make a slurry. The extract was then placed into a Vacuum Liquid Chromatography (VLC) column (5.0 cm × 120 cm), after being dried and slurred, for fractionation, and the obtained fractions were placed into column chromatography for final purification. The VLC column was packed with 350 g of silica gel 60 (230–400 mesh). The fractionation was carried out according to the following sequence: 0–100% gradients of EtOAc in n-hexane (by increasing 10.0% of EtOAc each time); 0–100% gradients of MeOH in EtOAc (by adding 5.0% of MeOH each time). Two hundred and fifty mL of elutes were always collected, and the solvents were dried with an evaporator under reduced pressure. Fractions that produced comparable spots were combined and concentrated until dry. The lovastatin-containing fraction underwent further chromatography over an open column (1.0 cm × 100 cm), which was packed with 60 g of silica gel 60 (70–230 mesh), and eluted isocratically with a n-hexane-EtOAc 20:80 solvent system. Twenty-five mL of elutes were harvested and spotted on TLC (mobile system: EtOAc/n-hexane; 80:20). Fractions that contained the most lovastatin were combined, dried, slurred, and uploaded over a second column (0.5 cm × 25 cm) that was packed with 15 g of silica gel 60, and the solvent system n-hexane-acetone 50:50 was used for elution. Fifteen mL of elutes were collected, subjected to TLC, and those containing a pure compound were combined.

### 4.11. LC-MS/MS Analysis

The assay was conducted at the Center of Drug Discovery Research and Development, Faculty of Pharmacy, Ain Shams University, Cairo, Egypt. The analysis was carried out using a LC-MS/MS system (Nexera with LC-MS-8045, Shimadzu Corporation, Kyoto, Japan) composed of HPLC (Nexera LC-30AD) coupled to a triple quadrupole mass spectrometer (Nexera with LC-MS-8045, Shimadzu Corporation, Kyoto, Japan). The HPLC was equipped with an auto-sampler (SIL-30AC), a temperature-controlled column oven (CTO-20AC), and a photodiode array detector (LC-2030/2040) with detection wavelengths of 235, 254, and 280 nm with λ max absorption at 220–400 nm. The LC-MS/MS was equipped with an RP-C18 UPLC column (Shimpack; 2 × 150 mm) possessing a particle size of 2.7 µm. The gradient elution system was composed of Acetonitrile (ACN) and 0.1% (*v*/*v*) formic acid in H_2_O. The elution was performed according to the following sequence: 0–2 min by 10% ACN, 2–26 min by 10–80% ACN, and finally 26–33 min by 100% ACN, with a flow rate of 0.2 mL/min. A positive mode was operated during LC-MS/MS with electrospray ionization (ESI). LC-MS/MS data were collected and processed by LC mass software, version 3.05 (Shimadzu Corporation, Kyoto, Japan). 

### 4.12. Spectroscopic NMR

The assay was performed at the Micro-analytical Unit (MAU), Faculty of Pharmacy, Cairo University, Giza, Egypt. ^1^H and ^13^C-NMR spectra were measured on a Bruker Avance III HD 400 and 100 MHz spectrometer, respectively (Bruker Biospin, Rheinstetten, Germany), with Linux CentOS release 5.11 and NMR software Topspin 3.2 pl 6, using tetramethylsilane (TMS) as an internal reference standard. HR-ESI-MS data were recorded on a LTQ Orbitrap XL spectrometer (Thermo Fisher Scientific; Waltham, MA, USA).

### 4.13. Thin Layer Chromatograph (TLC)

TLC was carried out on silica gel-precoated plates (F_254_). The plates were subjected to UV examination (at 365 and 254 nm), and visualization was performed by spraying them with 10% (*v*/*v*) H_2_SO_4_ in methanol, drying them with a hot air dryer, and then heating them to 110 °C. Optical density data were obtained using an ELISA reader (start fax-2100/USA). 

### 4.14. DPPH Radical Scavenging Activity

Following the Yen and Duh [108] methodology, a freshly made methanol solution containing 0.004% (*w*/*v*) of the 2,2-diphenyl-1-picrylhydrazyl (DPPH) radical was chilled at 10 °C in the dark. Separately, lovastatin concentrations of 100, 50, 25, 12.5, 6.25, 3.125, 1.56, and 0.78 µM in a methanol solution were used for the pure-produced lovastatin sample and the lovastatin standard. A 40 µL aliquot of the sample-containing methanol solution was mixed with 3 mL of the DPPH solution, and the absorbance was measured at 515 nm (Spectronic 1201, Milton Roy; Canada). The reduction in absorbance was continuously monitored until the absorbance stabilized, with data collected at 1 min intervals for 16 min. The absorbance of the DPPH radical in the absence of an antioxidant as well as that of ascorbic acid, a reference molecule, were both measured. The DPPH radical’s percentage inhibition (PI) was calculated according to Equation (2):

PI = (AC − AT) × 100(2)

where AC = control absorbance at t = 0 min and AT = sample absorbance + DPPH at t = 16 min. The 50% inhibitory concentration (IC_50_), the concentration required to inhibit DPPH radical by 50%, was estimated from graphic plots of the dose response curve. The experiment was conducted in triplicate.

### 4.15. Antimicrobial Activity of Lovastatin Produced by A. terreus AUMC 15760 

The antimicrobial potential of lovastatin produced by *A. terreus* AUMC 15760, compared to the standard lovastatin (Sigma-Aldrich), was tested against three fungi (*C. albicans* AUMC 13415, *C. glabrata* AUMC 13412, and *C. krusei* AUMC 13420) and three strains of *E. coli* ATCC 8739, *S. aureus* ATCC 6538, and *S. epidermidis* ATCC 12228. Both drugs were used at concentrations of 5, 2.5, 1.25, 0.62, and 0.31 mg/mL. Nystatin (50 mcg/disc) and Piperacillin/Tazobactam 10:1 (110 µg/disc) were used as antifungal and antibacterial reference drugs, respectively. The test organisms were grown in 24-h-old cultures, and spore suspensions (10^8^ CFU/mL = 0.5 McFarland standard solution) were employed to inoculate Petri plates. Using sterile cotton swabs and a sterile Petri plate filled with either Sabouraud’s dextrose agar (SDA) for *Candida* species or nutritional agar (NA) for bacteria, the bacterial and fungal spore suspensions were evenly distributed. Using the well diffusion method [109], the five wells (5 mm-diameter holes cut in the agar gel) each received a 50 µL addition of the tested drug. The plates were incubated for 24 h at 37 ± 1 °C for bacteria and *Candida* under aerobic conditions. After incubation, the inhibition of bacterial and fungal growth was measured in mm. Tests were performed in triplicate.

### 4.16. Statistical Analysis

Data were subjected to analysis of variance (ANOVA: one-factor with replication) followed by the Duncan’s multiple range test [110].

## 5. Conclusions

In this study, the ITS region was sequenced in order to identify three strains of *A. terreus*. GC-MS was used to determine the ability of the three strains to generate lovastatin in SSF using wheat bran. The potent strain produced the most lovastatin from sugarcane bagasse under ideal circumstances. The generated lovastatin was purified using column chromatography and identified using spectroscopic analyses, including optical density, LC-MS/MS, ^1^H, ^13^C-NMR, and HR-ESI-MS. The purified compound had DPPH activity and an IC_50_ that was almost identical to that of the reference drug. The purified drug’s antimicrobial property was judged to be effective against *C. albicans*, *C. glabrata*, *S. aureus*, and *S. epidermidis*. The plan for the future is to use various lignocellulosic residues to produce commodities with added value, significant secondary metabolites, enzymes, anticancer compounds, and growth-promoting agents, all of which are crucial for sustainable development.

## Figures and Tables

**Table 1:** ^1^H and ^13^C-NMR spectroscopic data of lovastatin (lactone form) measured in CD_3_OD (400 and 100 MHz, respectively).

No	*δ*_H_, (*J* in H_z_)	*δ*_C_, Type	No	*δ*_H_, (*J* in H_z_)	*δ*_C_, Type
1	5.39, 1H, m	69.5 d	13	4.64, 1H, m	78.0 d
2a	1.98, 1H, m	33.6 t	14a	1.95, 1H, m	36.6 t
2b	1.82, 1H, m		14b	1.79, 1H, m	
3	2.45, 1H, m	28.8 d	15	4.26, 1H, m	63.3 d
4	5.52, 1H, br.t (2.8)	130.3 d	16a	2.73, 1H, dd (4.8, 16.8)	39.1 t
5	-	129.5 s	16b	2.56, 1H, ddd (1.6, 4.8, 16.8)	
6	6.00, 1H, d (9.6)	129.8 d	17	-	173.4 s
7	5.80, 1H, dd (6.0, 9.6)	133.9 d	18	0.94, 3H, d (7.2)	14.1 q
8	2.42, 1H, m	31.9 d	19	1.10, 3H, d (7.2)	23.3 q
9	1.75, 1H, m	37.9 d	1′	-	178.2 s
10	2.38, 1H, dd (6.8, 7.2)	38.5 d	2′	2.36, 1H, m	42.8 d
11a	1.52, 1H, m	25.1 t	3a′	1.68, 1H, m	27.9 t
11b	1.45, 1H, m		3b′	1.50, 1H, m	
12a	1.91, 1H, m	34.0 t	4′	0.93, 3H, dd (2.0, 7.2)	12.1 q
12b	1.37, 1H, m		5′	1.12, 3H, d (7.2)	16.6 q

**Table 2:** The antimicrobial potential of the purified lovastatin (mean ± SD, *n* = 3 *) produced by *A. terreus* AUMC 15760 on three strains of fungi and three strains of bacteria, compared to antifungal Nystatin (NS) and antibacterial Piperacillin/Tazobactam (P/T).

Tested Organisms	Purified Lovastatin (mg/mL)					NS (50 mcg)	P/T (110 µg)
	5	2.5	1.25	0.62	0.31		
*C. albicans*	10.5 ± 0.2 ^c^	9.2 ± 0.2 ^e^	0 ^g^	0 ^g^	0 ^g^	21.0 ± 1.6 ^b^	-
*C. glabrata*	8.7 ± 0.4 ^d^	0 ^g^	0 ^g^	0 ^g^	0 ^g^	22.43 ± 1.2 ^a^	-
*C. krusei*	0 ^f^	0 ^f^	0 ^f^	0 ^g^	0 ^g^	16.2 ± 0.8 ^d^	-
*E. coli*	0 ^f^	0 ^f^	0 ^f^	0 ^f^	0 ^f^	-	15.3 ± 0.2 ^e^
*S. aureus*	20.3 ± 0.6 ^c^	15.6 ± 0.2 ^e^	11.5 ± 0.7 ^h^	0 ^f^	0 ^f^	-	18.0 ± 0.3 ^a^
*S. epidermidis*	19.4 ± 0.5 ^e^	15.3 ± 0.1 ^d^	11.4 ± 0.4 ^f^	0 ^f^	0 ^f^	-	15.36 ± 0.4 ^b^

* At the 0.05 level of probability, means in a column with the same letters are not statistically different. Averages having the same letter are not significant at the 5% level, according to the Least Significant Difference Test.

## Data Availability

All data related to this manuscript are incorporated only into the manuscript.

## Supplementary Materials

The following supporting information can be downloaded at: https://www.mdpi.com/article/10.3390/molecules28104048/s1, Table S1: GC–MS spectral analysis of the chemical compounds detected in the methanol extract of *A. terreus* AUMC 15760; Figure S1: GC-MS chromatogram of the methanol extract of *A. terreus* AUMC 15760; Table S2: GC-MS spectral analysis of the chemical compounds detected in the methanol extract of *A. terreus* AUMC 15762; Figure S2: GC-MS chromatogram of the methanol extract of *A. terreus* AUMC 15762; Table S3: GC-MS spectral analysis of the chemical compounds detected in the methanol extract of *A. terreus* AUMC 15763; Figure S3: GC-MS chromatogram of the methanol extract of *A. terreus* AUMC 15763; Figure S4: A. LC chromatogram of the fraction Fr. 8-S5. B. Positive ion LC-MS/MS chromatogram of detected lovastatin in the fraction Fr. 8-S5 produced by *A. terreus* AUMC 15760; Figure S5. A. Total ion chromatogram. B. Electron Spray Ionization Mass spectrum of the pure isolated lovastatin; Figure S6: ^1^H-NMR spectrum of lovastatin measured in (CD_3_OD, 400 MHz); Figure S7: ^13^C-NMR spectrum of lovastatin measured in (CD_3_OD, 100 MHz); Figure S8: Effect of the purified lovastatin on: (A), *C. albicans* (B), *C. glabrata* (C), *S. aureus* (D), and *S. epidermidis* (at 1: 5 mg/mL; 2: 2.5 mg/mL; 3: 1.25 mg/mL; 4: 0.62 mg/mL; and 5: 0.31 mg/mL) compared to Nystatin (NT) and Piperacillin/Tazobactam (P/T).
