# Supplementary material for: Exploitation of Sugarcane Bagasse and Environmentally Sustainable Production, Purification, Characterization, and Application of Lovastatin by Aspergillus terreus AUMC 15760 under Solid-State Conditions
Source: Molecules. 2023 May 12;28(10):4048. doi: 10.3390/molecules28104048 (PMC10222103; doi:10.3390/molecules28104048)
Supplement: Supplementary file 1 [file molecules-28-04048-s001.zip › molecules-2341621-supplementary.pdf]

molecules
MDPI

Supplementary Material

Exploitation of Sugarcane Bagasse and Environmentally Sustainable Production, Purification, Characterization, and Application of Lovastatin by *Aspergillus terreus* AUMC 15760 under Solid-State Conditions

Ahmed M. A. A. Ramadan [1], Reda M. Shehata [1,2], Hussein H. EL-Sheikh [1,2], Fuad Ameen [3,*], Steven L. Stephenson [4], Sabry A. H. Zidan [5] and Osama A. M. Al-Bedak [6,*]

[1] Department of Botany & Microbiology, Faculty of Science, Al Azhar University, Cairo 11511, Egypt; micro_boy95@yahoo.com (A.M.A.A.R.); redamostafa.18@azhar.edu.eg (R.M.S.); husseinhosny.221@azhar.edu.eg (H.H.E.-S.)
[2] The Regional Center for Mycology and Biotechnology (RCMB), Al Azhar University, Cairo 11511, Egypt
[3] Department of Botany & Microbiology, College of Science, King Saud University, Riyadh 11451, Saudi Arabia
[4] Department of Biological Sciences, University of Arkansas, Fayetteville, AR 72701, USA; slsteph@uark.edu
[5] Department of Pharmacognosy, Faculty of Pharmacy, Al-Azhar University, Assiut Branch, Assiut 71524, Egypt
[6] Assiut University Mycological Centre, Assiut 71511, Egypt
* Correspondence: fuadameen@ksu.edu.sa (F.A.); osamaalbedak@science.au.edu.eg (O.A.M.A.-B.)

Table S1. GC–MS spectral analysis of the chemical compounds detected in the methanol extract of *A. terreus* AUMC 15760.

No.	Retention time (min)	Name of the compound	Molecular Weight	Molecular Formula
1	10.844	(2Z)-3-Methyl-2-decene	154	$C_{11}H_{22}$
2	12.018	(Z,Z)-9,12-Octadecadienoic acid	280	$C_{18}H_{32}O_2$
3	11.569	2-Methoxyphenazine	210	$C_{13}H_{10}N_2O$
4	20.835	4,5,6,7-Tetramethyl-1H-benzimidazole	174	$C_{11}H_{14}N_2$
5	9.442	DL-Arabinitol	152	$C_5H_{12}O_5$
6	**21.672**	**Lovastatin**	**404**	$\mathbf{C_{24}H_{36}O_5}$
7	11.299	Palmitic acid	256	$C_{16}H_{32}O_2$
8	**28.669**	**Simvastatin**	**418**	$\mathbf{C_{25}H_{38}O_5}$

9	10.985	Sorbitol	182	$C_6H_{14}O_6$
10	9.706	Xylitol	152	$C_5H_{12}O_5$

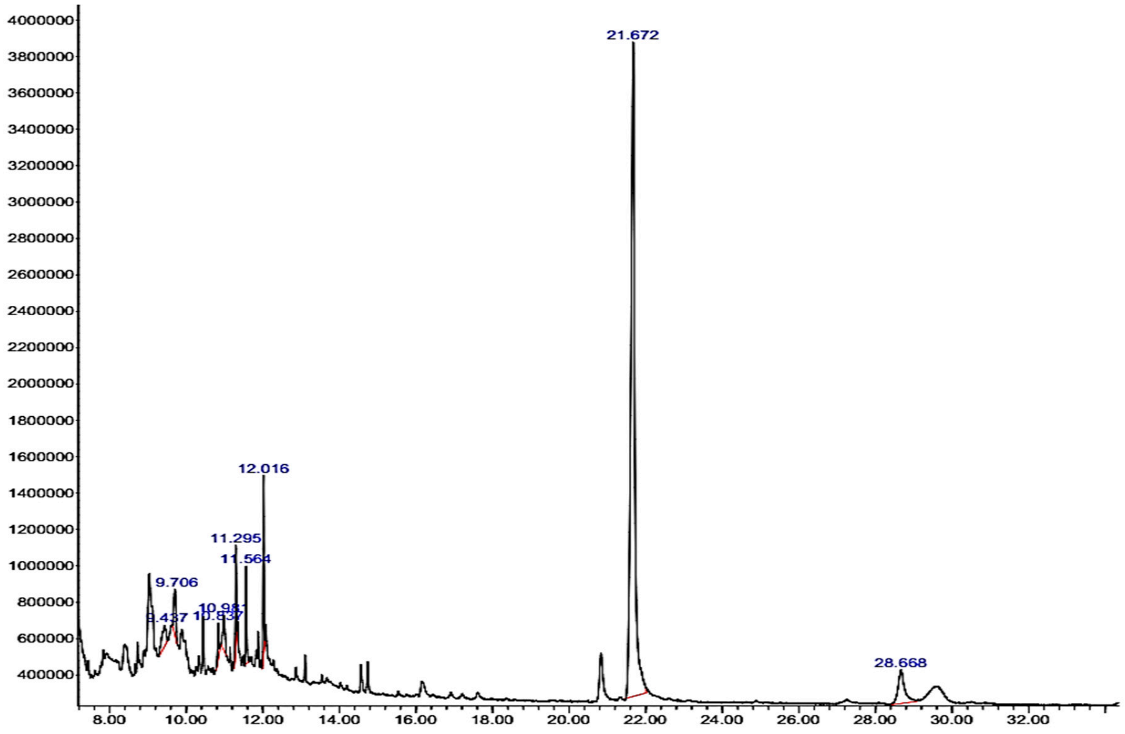

Figure S1. GC-MS chromatogram of the of methanol extract of *A. terreus* AUMC 15760.

molecules MDPI

Table S2. GC–MS spectral analysis of the chemical compounds detected in the methanol extract of *A. terreus* AUMC 15762.

**No.**	**Retention time (min)**	**Name of the compound**	**Molecular Weight**	**Molecular Formula**
1	10.444	4-((1E)-3-Hydroxy-1-propenyl)-2-methoxyphenol	180	$C_{10}H_{12}O_3$
2	11.569	4-Nitro-2,6-dimethylphenol	167	$C_8H_9NO_3$
3	12.024	cis, cis-Linoleic acid	266	$C_{17}H_{30}O_2$
4	9.436	Deoxyribose	134	$C_5H_{10}O_4$
5	9.116	Mono trimethylsilyl acrylic acid	144	$C_6H_{12}O_2Si$
6	**21.673**	**Lovastatin**	**404**	$\mathbf{C_{24}H_{36}O_5}$
7	8.403	N-Ethyl-2-carbomethoxyazetidine	143	$C_7H_{13}NO_2$
8	9.608	Ribitol	152	$C_5H_{12}O_5$
9	11.665	Sorbitol	182	$C_6H_{14}O_6$
10	9.025	Xylitol	152	$C_5H_{12}O_5$

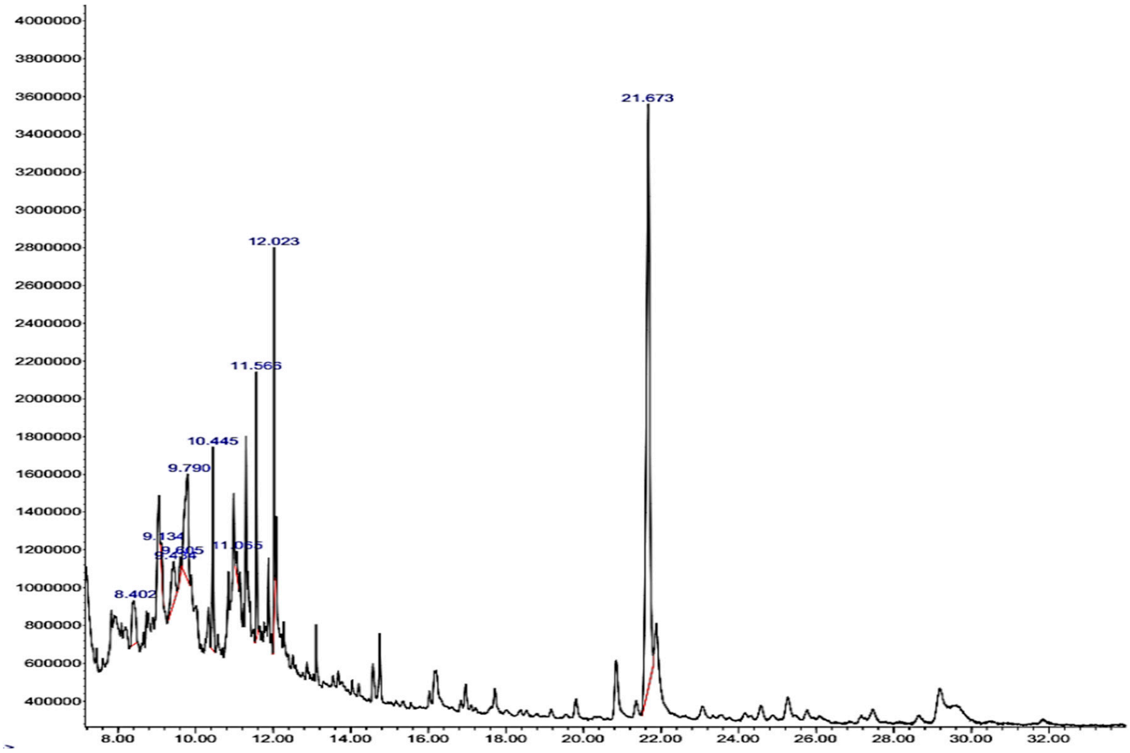

Figure S2. GC-MS chromatogram of the of methanol extract of *A. terreus* AUMC 15762.

molecules MDPI

Table S3. GC–MS spectral analysis of the chemical compounds detected in the methanol extract of *A. terreus* AUMC 15763.

**No.**	**Retention time (min)**	**Name of the compound**	**Molecular Weight**	**Molecular Formula**
1	29.191	2-Amino propanoic acid	88	$C_3H_7NO_2$
2	11.354	3-Isobutylhexahydropyrrolo[1,2-a]pyrazine1,4-dione	210	$C_{11}H_{18}N_2O_2$
3	12.024	cis, cis-Linoleic acid	266	$C_{17}H_{30}O_2$
4	20.854	4,5,6,7-Tetramethyl-1H-benzimidazole	174	$C_{11}H_{14}N_2$
5	9.116	N-Ethyl-2-carbomethoxyazetidine	143	$C_7H_{13}NO_2$
6	**21.709**	**Lovastatin**	**404**	$\mathbf{C_{24}H_{36}O_5}$
7	11.299	Palmitic acid	256	$C_{16}H_{32}O_2$
8	**28.687**	**Simvastatin**	**418**	$\mathbf{C_{25}H_{38}O_5}$
9	10.936	Sorbitol	182	$C_6H_{14}O_6$
10	9.054	Trans-Crotyl alcohol, tert-butyldimethylsilyl ether	186	$C_{10}H_{22}OSi$

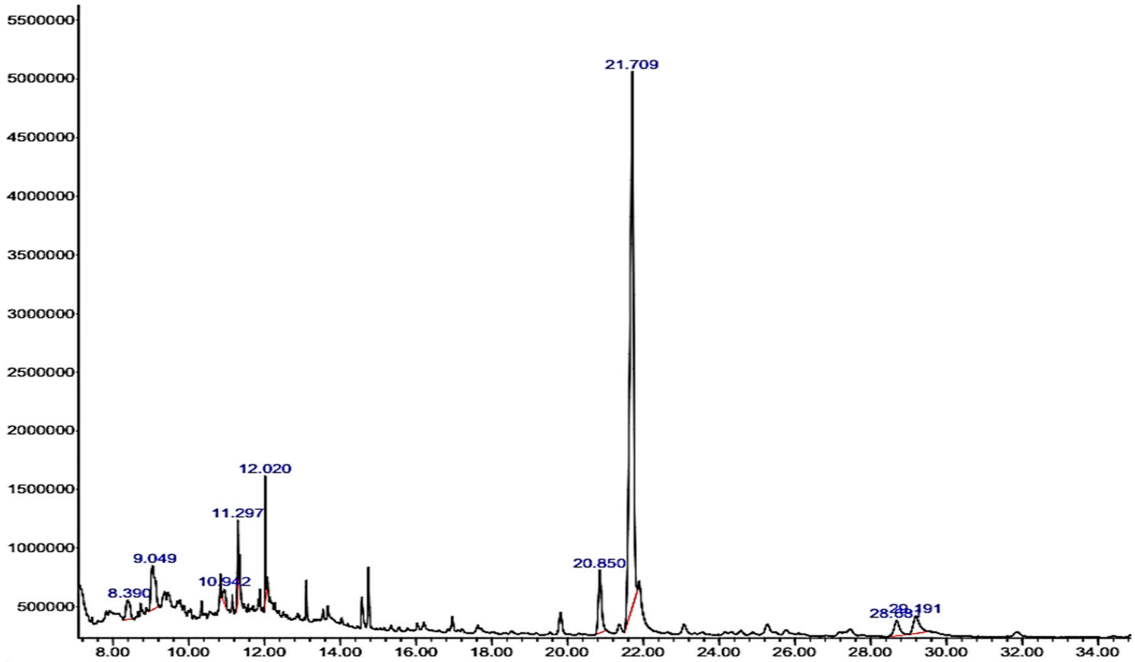

Figure S3. GC-MS chromatogram of the of methanol extract of *A. terreus* AUMC 15763.

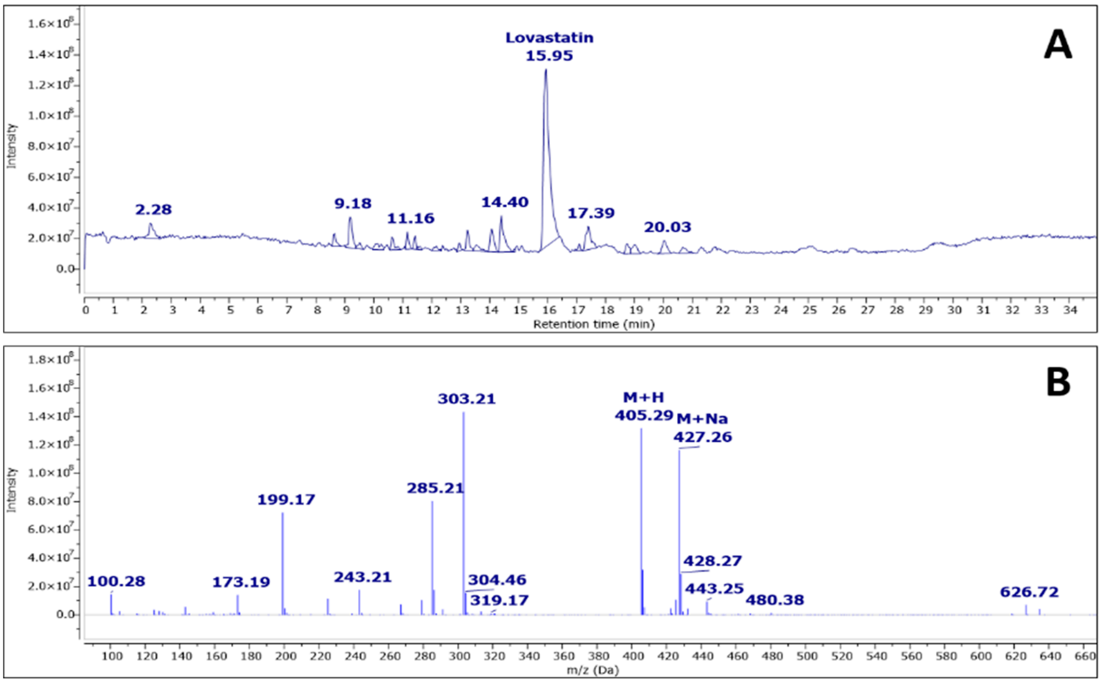

Figure S4. (A), LC chromatogram of the fraction Fr. 8-S5. (**B),** Positive ion LC-MS/MS chromatogram of detected lovastatin in the fraction Fr. 8-S5 produced by *A. terreus* AUMC 15760.

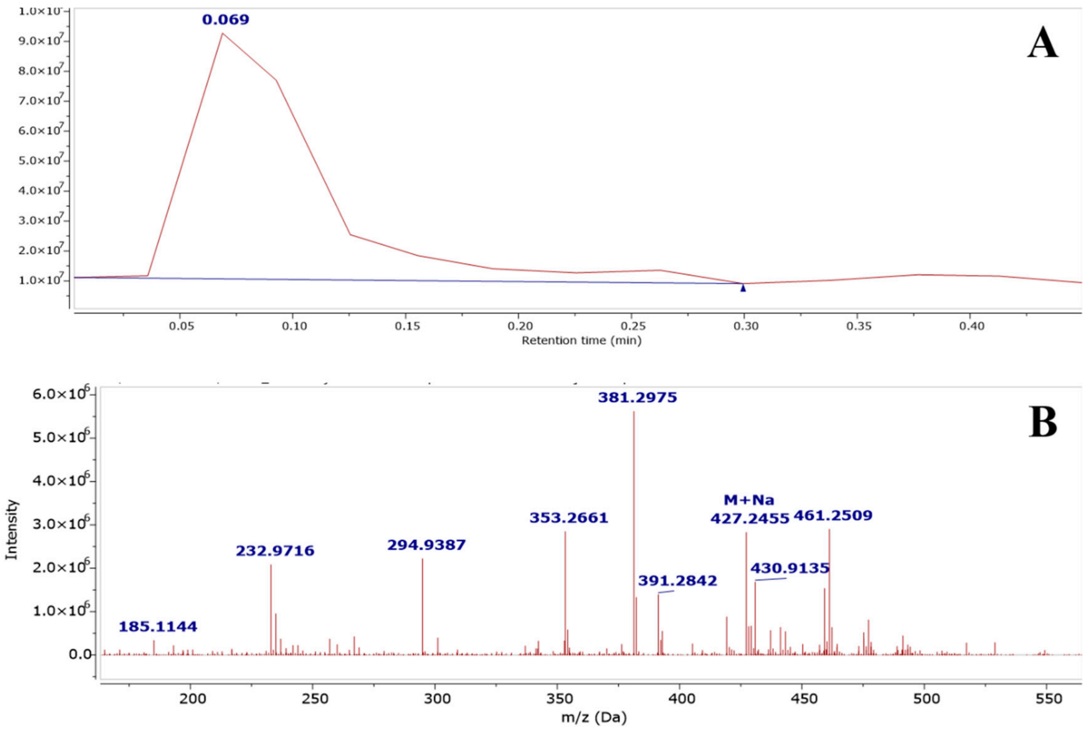

Figure S5. (A), Total ion chromatogram. **(B)**, Electron Spray Ionization Mass spectrum of the pure isolated lovastatin.

molecules

MDPI

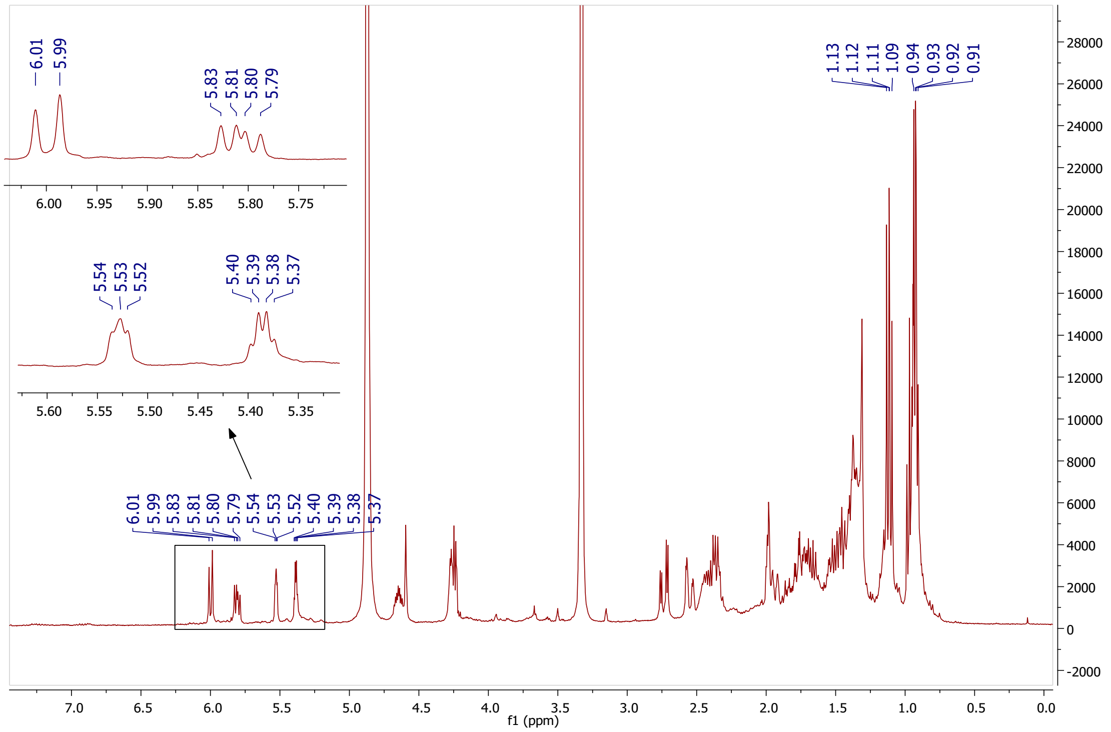

Figure S6. ^{1}H-NMR spectrum of lovastatin measured in (CD_3OD, 400 MHz).

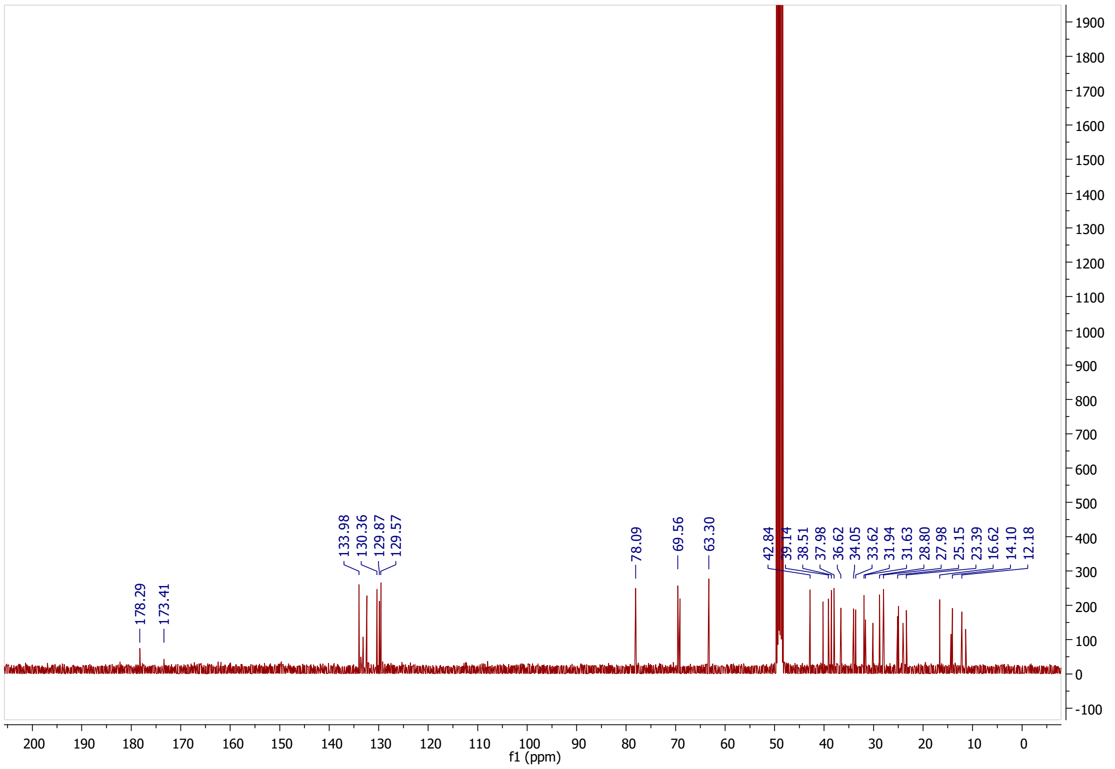

Figure S7. ^{13}C-NMR spectrum of lovastatin measured in (CD_3OD, 100 MHz).

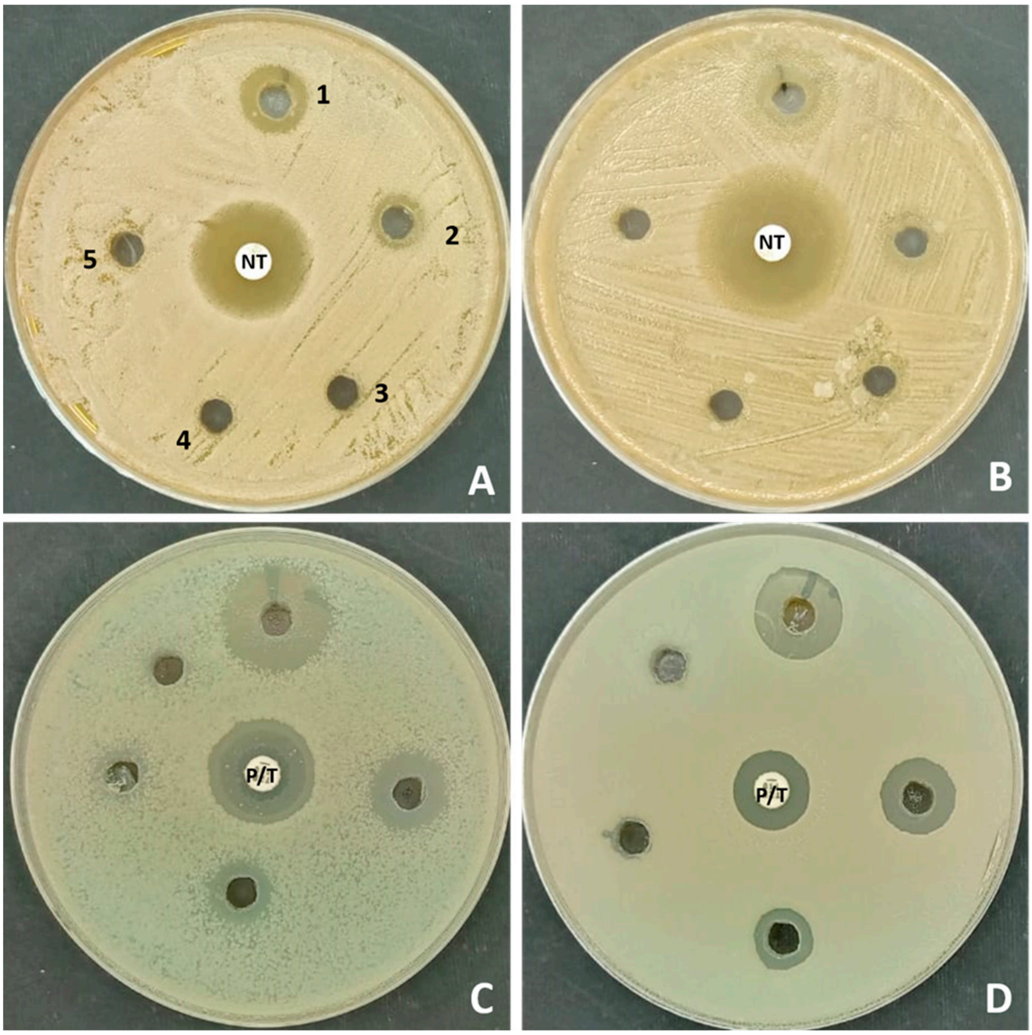

Figure S8. Effect of the purified lovastatin on: **(A),** *C. albicans* **(B),** *C. glabrata* **(C),** *S. aureus* **(D),** *S. epidermidis* (at 1: 5 mg/mL; 2: 2.5 mg/mL; 3: 1.25 mg/mL; 4: 0.62 mg/mL and 5: 0.31 mg/mL) compared to Nystatin (NT) and Piperacillin/Tazobactam (P/T).
